# Retrospective analysis of the effect of breast surgery on body posture in patients with early-stage breast cancer after cancer treatment (VENUS study)(Breast cancer and body posture)

**DOI:** 10.3906/sag-1912-22

**Published:** 2021-04-30

**Authors:** Özgür TANRIVERDİ, Ozan Ahmet ÇETİN, Ali ALKAN

**Affiliations:** 1 Department of Medical Oncology, Faculty of Medicine, Muğla Sıtkı Koçman University, Muğla Turkey; 2 Department of Internal Medicine, Faculty of Medicine, Muğla Sıtkı Koçman University, Muğla Turkey

**Keywords:** Breast cancer, body posture, thoracic kyphosis, Cobb’s angle

## Abstract

**Background/aim:**

The aim of this study was to determine whether breast surgery changes body posture in patients with early-stage breast cancer.

**Materials and methods:**

Study variables include age, side and localization of the tumor in the breast, applied breast surgery, axillary interference, pathological tumor size, axillary lymph node metastasis, body mass index, bone density, adjuvant therapies, and histological type. Thoracic kyphosis angle due to the anatomically affected primary region to detect changes in body posture and Cobb’s method were used to measure this.

**Results:**

There was a statistically significant difference in the mean Cobb’s angle between the follow-up times of 57 patients (P < 0.001), with a cumulative increase in the Cobb’s angle from baseline to the second year. As the age of the diagnosis progressed, the Cobb’s angle increased significantly at 2 years when compared to baseline (r = 0,616, P < 0,001). In terms of baseline, the higher the BMI level in the 2nd year, the higher the Cobb’s angle in the 2nd year as compared to the baseline (r = 0,529, P < 0,001).

**Conclusion:**

It was concluded that the increase in thoracic kyphosis in patients with breast cancer should be examined psychosocially. The study should be supported by a larger number of patients.

## 1. Introduction

Breast cancer is the most common type of malignancy in women, comprising 23% of all women’s cancer. Although there is a progressively growing data about screening, diagnosis, and treatment in the last decade, breast cancer is the second leading cause of death among women [1]. Surgery is the primary curative treatment of breast cancer [1]. The surgical approach, either modified radical mastectomy (MRM) or breast-conserving surgery (BCS), is decided according to the patient’s breast size, size of the mass, its location, axillary lymph node status, and also the patient’s preferences. In addition, sentinel lymph node biopsy is an option performed as a routine approach in the clinical practice to avoid unnecessary axillary dissections [1–3].

Although there has been improvement in surgical techniques and breast-conserving approaches, breast surgery has some inevitable complications [4]. Lymphedema is one of the complications of breast surgery and axillary lymph node dissection [3]. Lymphedema is an important morbidity, and it has negative effects on the quality of life of the patients. The postmastectomy pain syndrome is also another problem defined as a neuropathic pain resulting from the trauma of surgery. In addition, significant psychosocial problems may occur in breast cancer patients due to loss of a symbol of femininity and postmastectomy pain syndrome. Therefore, the current approach is to perform reconstructive surgery in suitable patients to avoid those physical and psychosocial problems [5–7].

There are limited data about the effects of breast surgery on body posture. The findings of our study can provide valuable data to the literature, and also they can lead to new studies in this field. During out-patient clinic visits, based on the observation of their sitting positions, it has been concluded that it is a behavior of hiding the operated breast; however, there have been no studies based on scientific data. Considering social and personal values, it may be caused by the loss of feminine identity and self. As there are not enough data on psychosocial issues of cancer patients during their treatment and follow-up process, the hypothesis based upon this observation may be considered consistent.

In addition to numerous long-term complications of breast surgery, body posture, and related abnormal sitting and standing postures have drawn attention in the last few years. There are limited data showing a change in posture of breast cancer survivors, associated with mastectomy and lymphedema [5–7]. These studies were heterogeneous, and different methods were used. Thus, there is a gap in the field of data. 

The aim of the study was to analyze the postural changes related with mastectomy and to identify the factors causing postural changes.

## 2. Materials and methods

This study was conducted as a retrospective study. 

### 2.1. Patients and methods

The histopathologically proven breast cancer patients diagnosed between 2011 July and 2018 December were analyzed. All the patients in remission more than 18 years of age and the ones having staging thoracic computerized tomography (CT), and follow-up CT in the 1st, 2nd, 3rd, 4th, and 5th years, were included in the study. The patients who had vertebral surgery before for any reason, who had ankylosing spondylitis before or vertebral involvement of rheumatoid and severe scoliosis, who had reconstructive breast prosthesis after mastectomy, who had recurrence, and who had lymphedema were excluded. 

The medical records of breast cancer survivors were analyzed retrospectively. As research variables, demographic features (age, height, weight, body mass index), clinical and histological characteristics (localization of primary tumor in the breast, histopathological type, type of surgery, number of lymph nodes dissected, number of metastatic lymph nodes, axillary nodal status, the size of pathological tumor, the drugs used in adjuvant therapy, history of adjuvant radiotherapy, the bone mineral density), and thoracic kyphosis measurements were done. 

To analyze the thoracic kyphosis, we used the CT scans performed for routine follow-up. The change in posture was evaluated by calculating Cobb’s angles (CAN). The CAN is an angle of curvature measured by drawing lines parallel to the upper border of the upper vertebral body and the lower border of the lowest vertebra of the structural curve, then erecting perpendiculars from these lines to cross each other, the angle between these perpendiculars is the CAN (Figure1A–1C) [5–7]. These computed tomography images are examples of how the CAN is measured. To assess the CAN, we used the sagittal views of CT scans. In sagittal view, for the thoracic spine area between T4 and T12, for lumbar region L1 and L5, and for thoracolumbar junction between T11 and L12 are ideal for the lines used for CAN measurement [5–7]. Kyphosis is defined as positive, whereas lordosis angle as negative. In many studies, it is stated that for increase in thoracic kyphosis, CAN is forty and above. It is acknowledged that curve in dorsal spine is normally between 20° and 40° [5–7]. The images in Figure 1 show the CAN of one patient (48 years, female patient, MRM) included in our study in tomography 1 month after surgery (Figure 1A), 1 year (Figure 1B) and 2 years (Figure 1C) after surgery and the change of this measurement by years.

**Figure:**
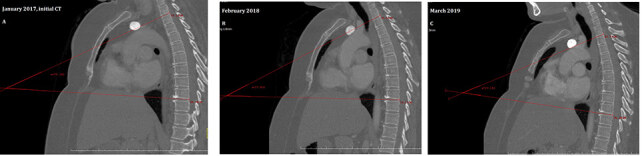
Demonstration of Cobb’s angle measurement method with computed tomography images of one of our patients in this study: 1 month after surgery (A), 1 year (B) and 2 years (C) after surgery.

### 2.2. Statistical analysis

Baseline characteristics of the patient group were described using means + SD for continuous variables, and frequencies and proportions for dichotomous and categorical variables. The distribution of continuous numerical variables was tested with the Shapiro–Wilk test and variant homogeneity with the Levene test. Differences between continuous variables were assessed with the Student t-test and nonparametric tests for repeated measures (the Friedman test). The chi-square or Fisher’s exact tests were used to compare categorical variables. The correlation of continuous numerical variables was tested by Spearman’s correlation. The incidences of thoracic kyphosis in different evaluation periods were analyzed with the McNemar test. Multivariate analysis was performed using a logistic regression model. The factors associated with thoracic kyphosis in univariate analysis (P > 0.10) were included in the multivariate analysis. 

All analyses were performed using SPSS 17.0 for Windows (IBM Corp., Armonk, NY). P-values less than 0.05 were considered statistically significant.

## 3. Results

The medical records of 967 breast cancer patients were analyzed retrospectively. Among these patients, 57 patients met the inclusion criteria. 57 patients with a mean age of 55.6 ± 12.5, ranging between 31 and 79 years were evaluated. The median follow-up time was 43.6 (range: 22.8–65.4). The BMI was 27.4 ± 1.8, and most of them were overweight (84%). Of the study population, 86% were operated with MRM, and 93% had axillary dissection. The median number of dissected lymph nodes was 15 (range: 1–33). Most of the tumors were located in the left breast (53%) and upper outer quadrant (51%), and invasive ductal carcinoma was present in 91% of the cases (Table 1). 

**Table 1:** Demographic and clinical features of patients.

Features	Findings
Age (years); mean ± standard deviation	55.6 ± 12.5
Body mass index (kg/m2); n (%)	
Normal weight	3 (5)
Overweight	48 (84)
Class 1 obesity	6 (11)
DEXA; n (%)	
Normal	13 (28)
Osteopenia	32 (68)
Osteoporosis	2 (4)
Type of surgery; n (%)	
BCS	8 (14)
MRM	49 (86)
Axillary surgery; n (%)	
Sentinel lymph node biopsy	4 (7)
Axillary dissection	53 (93)
Pathologic tumor size (mm); mean ± standard deviation	24.5 ± 2.7
Number of dissected lymph node; average (range)	15 (1-33)
Number of metastatic lymph node; average (range)	6 (0-23)
Basal cobb angle (°); mean ± standard deviation	33.6 ± 3.0
Follow-up time (months); average (range)	43.6 (22.8-65.4)
Localization; n (%)	
Right breast	27 (47)
Left breast	30 (53)
Localization in the breast; n (%)	
Upper outer quadrant	29 (51)
Upper inner quadrant	8 (14)
Lower outer quadrant	8 (14)
Lower inner quadrant	5 (9)
Areolar	6 (10)
Multifocal	1 (2)
Histological type; n (%)	
Invasive ductal carcinoma	52 (91)
Invasive lobular carcinoma	3 (5)
Tubular carcinoma	1 (2)
Infiltrating carcinoma	1 (2)
Axillary involvement; n (%)	
Present	27 (47)
Absent	30 (53)
Adjuvant chemotherapy; n (%)	49 (86)
Adjuvant radiotherapy; n (%)	37 (65)
Neoadjuvant chemotherapy; n (%)	10 (17)
Adjuvant endocrine treatment; n (%)	
Tamoxifen	7 (12)
Letrozole	14 (26)
Anastrozole	27 (47)
Absent	9 (15)

DEXA: dual energy X-ray absorptiometry; BCS: breast-conserving surgery; MRM: modified radical mastectomy.

The analysis of CAN revealed statistically significant changes within the first 2 years. The basal, 1st, and 2nd year CAN were 33.60 ± 2.97, 34.65 ± 3.57, and 36.19 ± 4.62, respectively (P < 0.001). The analysis of factors associated with CAN changes showed that there were positive correlations between ages (P < 0.001), BMI changes (P < 0.001), and CAN (Table 2). In addition, there was more increase in the BCS when compared to MRM (4.12 ± vs. 2.35 ± 2.24, P = 0.048) (Table 3). No correlations were observed between adjuvant radiotherapy and CAN measurements. Moreover, no statistically significant correlations were found with axillary dissection (Tables 2 and 3).

**Table 2:** Correlation coefficients between the changes in Cobb’s angle at the end of 2nd year according to the basal in cases followed for 2 years and other demographic-clinical features of the cases.

	Correlation coefficient	P-value †
Age	0.616	<0.001
Weight	0.028	0.838
Basal BMI	0.158	0.240
Follow-up time	-0.241	0.071
Tumor size	-0.040	0.772
Change in BMI	0.529	<0.001
Adjuvant radiotherapy	0.179	0.205
Axillary dissection	0.304	0.118

† Spearman’s rank-order correlation test. BMI: body mass index.

**Table 3:** The factors associated with increase in Cobb’s angle.

	n	Increase inCobb angle	P
DEXA			
Normal	13	2.08 ± 2.10	
Osteopenia/Osteoporosis	34	2.91 ± 2.54	0.298
Type of surgery			
BCS	8	4.12 ± 2.70	
MRM	49	2.35 ± 2.24	0.048
Adjuvant radiation treatment			
Present	37	2.36 ± 1.31	
Absent	20	2.04 ± 1.24	0.274
Axillary dissection			
Present	53	2.14 ± 1,11	
Absent	4	2.04 ± 1.06	0.138
Localization			
Right	27	2.85 ± 2.73	
Left	30	2.37 ± 2.01	0.445
General	57	2.60 ± 2.37	-

Data are shown as average ± standard deviation,† Student’s t-test. DEXA: dual energy X-ray absorptiometry; BCS: breast-conserving surgery; MRM: modified radical mastectomy.

In the cases followed for 5 years (n = 12), they indicate statistical comparisons in terms of change in CAN regarding the time. From basal to the 5th year, even though a cumulative increase was observed in CAN, there were no statistically significant differences (P = 0.120). The univariate analysis of factors associated with CAN in 5-year data showed that age (r = 0.957, P < 0.001) and basal BMI (r = 0.753, P = 0.005) had positive correlation with CAN. 

The multivariate analysis of factors associated with increased CAN is summarized in Table 4. Within 2 years, BMI and age were found to be associated with increase in CAN. Every 1 kg/m2 increase in BMI was associated with a 0.632° increase in the CAN (95% CI, 0.280–0.985, P < 0001). In addition, a 10-year increase in age was associated with 0.72° increase in CAN (95% CI, 0.31–1.13, P < 0.001). The 3-year data of 38 patients could be analyzed. Within 3 years, BMI, age, and type of surgery were found to be associated with increase in CAN. Every 1 kg/m2 increase in BMI was associated with a 0.836° increase in CAN (95% CI, 0.383–1.290, P < 0001). In addition, a 10-year increase in age was associated with 0.87° increase in CAN (95% CI, 0.30–1.44, P = 0.004). When compared to MRM, BCS resulted in a 2.112°) increase in CAN (95% CI, 0.141–4.082, P = 0.037). Within 4 years, the data of 26 patients could be analyzed. The age and type of surgery were found to be associated with increase in CAN. A 10-year increase in age was associated with 1.34° increase in CAN (95% CI, 0.46–2.22, P = 0.005). When compared to MRM, BCS resulted in a 3.311° increase in CAN (95% CI, 0.248–6.373, P = 0.035). The 5-year data of 12 patients could be analyzed. The age was the only statistical factor associated with CAN increase. A 10-year increase in age was associated with 3.20° increase in CAN (95% CI, 1.87–4.54, P < 0.001).

**Table 4:** Multivariate analysis of factors associated with Cobb’s angle in years.

	Regressioncoefficient	%95 confidence interval		t-statistics	P-value
		Lower limit	Upper limit		
Model 1 a					
Diagnosis age	0.072	0.031	0.113	3.528	<0.001
Monitoring times	–0.022	–0.055	0.010	–1.375	0.175
Change in BMI	0.632	0.280	0.985	3.598	<0.001
BCS	1.105	–0.146	2.356	–1.773	0.082
Model 2 b					
Diagnosis age	0.094	0.034	0.154	3.188	0.003
Pathologic tumor size	–0.020	–0.063	0.023	–0.927	0.361
Change in BMI	0.897	0.423	1.372	3.847	<0.001
Model 3 c					
Diagnosis age	0.136	0.041	0.232	2.971	0.007
Pathologic tumor size	–0.044	–0.103	0.015	–1.562	0.132
Change in BMI	0.940	–0.146	2.027	1.794	0.087
Model 4 d					
Diagnosis age	0.320	0.187	0.454	5.433	<0.001
Change in BMI	–0.315	–1.796	1.166	–0.481	0.642

a: Analysis done in cases followed for 2 years, b: analysis done in cases followed for 3 years, c: Analysis done in cases followed for 4 years, d: Analysis done in cases followed for 5 years.BMI: body mass index; BCS: breast-conserving surgery.

## Discussion

In the present study, we aimed to analyze the effects of MRM and BCS on the posture of breast cancer survivors. At 2 years, we found an increase in the thoracic kyphosis associated with age and body mass index. 

The first studies on the impact of surgery on posture were conducted in 1996 by Sliwinski et al. [8]. They concluded an association between mastectomy and spinal functional disorders. In addition, in 1998, Dobosz et al. showed that mastectomy resulted in changes in posture related with asymmetry in body [9]. The study by Rostkowska et al. [6] assessed the effects of mastectomy on posture by comparing cancer survivors with healthy controls. The showed distinct adverse changes in body posture of women after mastectomy in comparison with healthy women. The changes were mainly in asymmetry of trunk and shoulder girdle and greater forward leaning of the trunk. They also found the positive effects of exercise on the abnormal body posture. In addition, there are numerous studies showing mastectomy-related body asymmetry and kyphotic and lordotic angle changes [5–9]. However, the results of the data about the influence of the operated breast (right or left) are inconclusive [5–12]. Due to study design by Rostkowska et al. [6], it is difficult to test the association between mastectomy and body posture because there was only one measurement in the study population and the locations of the tumors were absent. In addition, other factors affecting posture and presence of lymphedema were missing. Haddad et al. [5] worked on breast cancer survivors who had lymphedema. They found that women with mastectomy presented with asymmetries and modifications in posture, and lymphedema seemed to worsen this condition. Additionally, they had deficits in range of motion in the shoulders on the operated side [5]. Similar results were confirmed by Malicka et al. [10]. 

Rahimi et al. [11] compared the thoracic kyphosis and lumbar lordosis in breast cancer survivors with healthy women. They used a 60-cm-long flexi-curve to measure the size of the curve in thoracic and lumbar spines, and they found that breast cancer survivors experience more increased thoracic kyphosis compared to healthy women. Jeong et al. [7] evaluated the effect of immediate breast reconstruction on body posture after surgery by analyzing spinal alignment with radiographic studies. They found a significant difference in the CAN between the preoperative and 2-year postoperative chest radiographs between the immediate breast reconstruction group and mastectomy group. The amount of change in postoperative spinal alignment was significantly smaller in the immediate breast reconstruction group compared with patients receiving only unilateral mastectomy without reconstruction (7). 

In our study, at 2 years, we found a statistically significant change in CAN. In addition, there was a positive correlation between age, BMI, and thoracic kyphosis. However, this association could not be demonstrated in the following years. This could be explained by the missing data and limited number of patients after 2 years of follow-up. Although we used a different technique when compared to the literature, 2-year postural changes were consistent with previous studies. Similar to the study by Jeong et al. [7], age and BMI were associated with an increase in CAN. The link between age and increased thoracic kyphosis has been reported. The studies in healthy adolescents have shown an increase in thoracic kyphosis and lumbar lordosis in overweight and obese individuals [5–7,12]. In addition, the impact of age on thoracic kyphosis has been reported [5–7]. Osteoporosis, degenerative changes, and increase in body weight are other factors related with increased thoracic kyphosis [5–10].

There were some inevitable limitations in our study. There were a limited number of patients appropriate for the analysis. Due to retrospective design, breast volumes and the presence of external breast prosthesis could not be included in the analysis. In addition, we could not perform psychosocial tests. The number of patients after 2 years of follow-up was also limited. This could have affected the results. 

In conclusion, this project based on the hypothesis regarding whether the behavior of hiding removed breast in sitting or standing position causes any change in posture or not presents significant data. In fact, it is considered to be an important and guiding study in order to determine factors having effects in case of any change in patients’ postures, to study the psychosocial effect and to understand the possible consequences of the situation (fracture risk, pain, etc.). This study may be supported by prospective studies in which there are more patients, psychosocial measurements are used, and a control group is determined.

## Informed consent

The study was approved by the institutional Clinic Research Ethics Committee from Muğla Sıtkı Koçman University, and the study was performed in accordance with the declaration of Helsinki.
